# A classical phenotype of Anderson-Fabry disease in a female patient with intronic mutations of the GLA gene: a case report

**DOI:** 10.1186/1471-2261-12-39

**Published:** 2012-06-08

**Authors:** Antonio Pisani, Massimo Imbriaco, Carmela Zizzo, Giuseppe Albeggiani, Paolo Colomba, Riccardo Alessandro, Francesco Iemolo, Giovanni Duro

**Affiliations:** 1 Department of Nephrology, University of Naples, Federico II, Italy; 2 Department of Radiology, University of Naples, Federico II, Italy; 3 Institute of Biomedicine and Molecular Immunology “A. Monroy”, National Research Council, Via Ugo La Malfa 153, 90146, Palermo, Italy; 4 Dipartimento di Biopatologia e Biotecnologie Mediche e Forensi, Sezione di Biologia e Genetica, University of Palermo, Palermo, Italy

**Keywords:** Fabry disease, α-galactosidase A, GLA, Globotriaosylceramide, High resolution melting

## Abstract

**Background:**

Fabry disease (FD) is a hereditary metabolic disorder caused by the partial or total inactivation of a lysosomal hydrolase, the enzyme α-galactosidase A (GLA). This inactivation is responsible for the storage of undegraded glycosphingolipids in the lysosomes with subsequent cellular and microvascular dysfunction. The incidence of disease is estimated at 1:40,000 in the general population, although neonatal screening initiatives have found an unexpectedly high prevalence of genetic alterations, up to 1:3,100, in newborns in Italy, and have identified a surprisingly high frequency of newborn males with genetic alterations (about 1:1,500) in Taiwan.

**Case presentation:**

We describe the case of a 40-year-old female patient who presented with transient ischemic attack (TIA), discomfort in her hands, intolerance to cold and heat, severe angina and palpitations, chronic kidney disease. Clinical, biochemical and molecular studies were performed.

**Conclusions:**

Reported symptoms, peculiar findings in a renal biopsy – the evidence of occasional lamellar inclusions in podocytes and mesangial cells – and left ventricular (LV) hypertrophy, which are considered to be specific features of FD, as well as molecular evaluations, suggested the diagnosis of a classical form of FD.

We detected four mutations in the GLA gene of the patient: -10C>T (g.1170C>T), c.370-77_-81del (g.7188-7192del5), c.640-16A>G (g.10115A>G), c.1000-22C>T (g.10956C>T). These mutations, located in promoter and intronic regulatory regions, have been observed in several patients with manifestations of FD. In our patient clinical picture showed a multisystemic involvement with early onset of symptoms, thus suggesting that these intronic mutations can be found even in patients with classical form of FD.

## Background

Fabry disease is a rare pathology caused by mutations in the gene encoding the enzyme α-galactosidase A, a lysosomal hydrolase which detaches the galactose bound in α from globotriaosylceramide (Gb3) during glycosphingolipid metabolism [1]. The alteration of this process leads to the accumulation of Gb3 inside lysosomes of different cell types, with consequent cellular damage, giving rise to the phenotype of FD.

Recent studies suggest that FD is under-diagnosed in the general population. Neonatal screening studies have found a surprisingly high incidence of genetic alterations, 1:3,100, among infants in Italy and 1:1,500 among newborn males in Taiwan [2,3]. In the latter study, 86% had an intronic mutation, c.936+919G>A, that is associated with an atypical form of FD which is characterized by residual enzyme activity [3].

The classical form of FD is characterized by acroparesthesias and angiokeratomas in childhood, severe skin manifestations in adolescence, and progressive organ involvement with renal failure, cardiac and cerebrovascular disease in adulthood [4].

Female patients have an extremely variable phenotype, ranging from no disease to more severe clinical forms, due to random X-chromosome inactivation (Lyonization) [5].

Atypical forms of FD are characterized by symptoms affecting a single, specific organ, especially the heart, kidney and brain [6,7]. These renal, cardiac, or cerebrovascular variants manifest later in life, and are often associated with residual enzyme activity. Cases that should be considered among the possible atypical variants of FD include those in which the coding regions of the gene are wild type but there are single nucleotide substitutions and small deletions present in the regulatory and intronic regions. Some of these mutations can be considered polymorphisms, since they are present in more than 1% in the general population.

This paper presents the results of a study conducted on a woman with clinical symptoms related to FD. In our patient the typical picture of FD with a multisystemic involvement, the early onset of symptoms and molecular evaluations suggest that the presence of mutations in promoter and regulatory intronic regions of the GLA gene can be found even in patients with classical form of FD.

## Case presentation

A 40-year-old female presented as an outpatient with chronic kidney disease. At 23 years old, she had been hospitalized for a TIA and was diagnosed with vasculitis. Occasionally, she had experienced discomfort in her hands and feet that was triggered by warm or cold temperatures. At age 27, several other TIA episodes occurred. When the patient was 37, she was hospitalized for an episode of severe angina and palpitations. At that time, a transthoracic echocardiography was performed that showed concentric LV hypertrophy, with an end-diastolic septal wall thickness of 14 mm, a normal ejection fraction of 53%, and impaired diastolic function; in addition, hypokinesia of the infero-lateral wall was observed. Further evaluation included a myocardial scintigraphy with Tc-99 m-MIBI that showed a persistent defect at the level of the infero-lateral region of the LV, consistent with the diagnosis of myocardial infarct. The patient suffered another TIA episode during the period of hospitalization. Furthermore, the patient presented proteinuria with value of 1.2 g/24 hrs, normal blood pressure (130/70 mmHg), serum creatinine levels of 2.3 mg/dl, a glomerular filtration rate of 35 ml/min, serum Urea of 71 mg/dl, serum albumin of 4.2 g/dl. Anti-nuclear antibodies (ANA), Coombs test, antibodies anti-platelets and anti-granulocytes were negative. The patient also reported that she had undergone two normal pregnancies. Her family medical history was negative.

**Figure 1:**
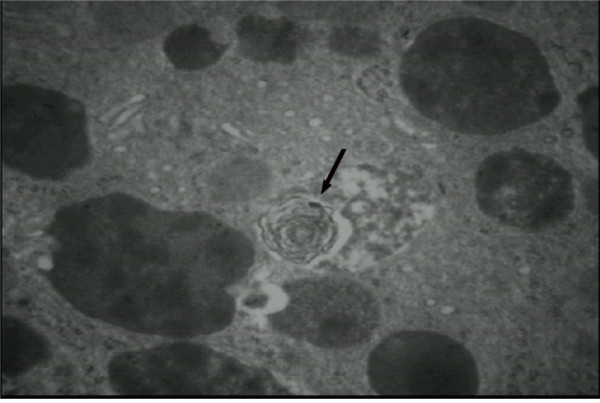
** Electron microscopy on a renal biopsy from a 40-year-old woman with FD.** Typical lysosomal inclusions in tubular cells are shown (arrow).

In order to evaluate the nature of the persistent proteinuria and the impaired renal function, the patient underwent a renal biopsy. Optical microscopy of the glomeruli (38–61) showed collapse in 4–6 of them and glomerular sclerosis in the others (data not shown). The interstitium showed a chronic inflammatory infiltrate and focal areas of tubular atrophy; residual tubules showed epithelial vacuolization and focal fibrosis. Immunofluorescence was negative for IgA, IgG, IgM, kappa and lambda chains, C3, C4, C1q, and fibrinogen. An electron microscopy study (Figure 1) showed several typical lysosomal inclusions in tubular cells, podocytes and mesangial cells, which are histological hallmark of FD [8].

**Figure 2:**
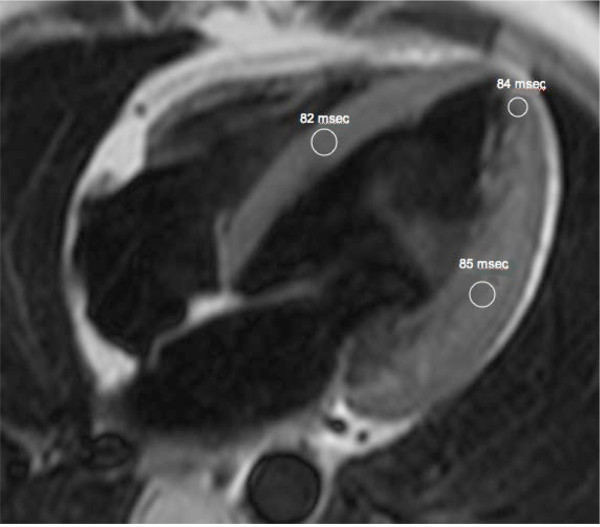
** Four-chamber horizontal long-axis T2-weighted black blood turbo spin-echo MR image.** White circles outline regions of interest in the mid septum, apex, and infero-lateral wall. MT2RTs were 82 milliseconds in mid septum, 84 milliseconds in apex, and 85 milliseconds in infero-lateral wall.

**Figure 3:**
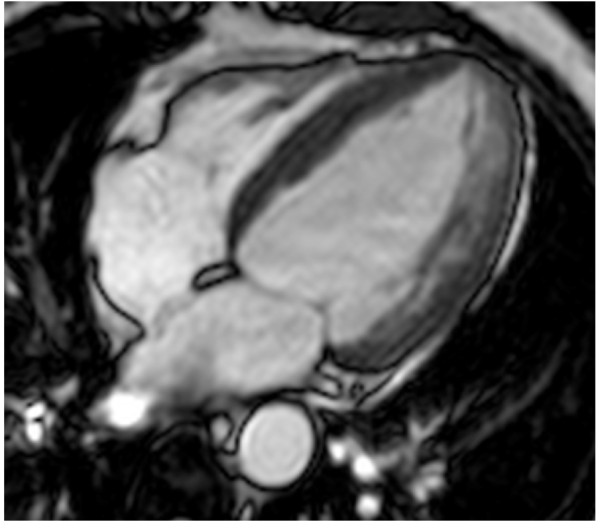
** Four-chamber horizontal long-axis balanced fast field echo image.** There is evidence of LV concentric hypertrophy.

To further characterize cardiac morphology, the patient underwent a cardiovascular magnetic resonance (CMR) study using a 1.5-T MRI system (Gyroscan Intera, Philips Medical Systems) equipped with high performance gradients. After a survey scan was obtained, a breath-holding T2-weighted black blood multiecho multishot turbo spin-echo (TSE) sequence with four different TEs was used to obtain images of the four-chamber horizontal long-axis plane for myocardial T2 relaxation time (MT2RT) measurements. LV four-chamber horizontal long-axis images were acquired using a breath-holding 3D balanced turbo field-echo multiphase multislice sequence; subsequently, biventricular short-axis images were obtained for evaluation of LV mass. MT2RT and LV mass were calculated as previously described [9,10]. There was a significantly prolonged MT2RT throughout the entire LV myocardium (Figure 2). A prolongation of MT2RT throughout the entire myocardium may be observed in patients with FD [9,10]. Researchers have shown that factors such as myocardial water and lipid alterations can lead to an abnormal prolongation of the MT2RT and to an increase in signal intensity [11]. Therefore, it is reasonable to presume that the prolonged MT2RT in all myocardial regions, as observed in this case, was related to the marked glycolipid deposition in the myocardium. Our patient showed an ejection fraction of 53%, LV mass of 128 g, inter-ventricular septal wall thickness of 14 mm and postero-lateral wall thickness of 15 mm (Figure 3).

As a result of the renal biopsy and cardiac MRI study findings, associated to the clinical history, suggesting FD, the patient underwent enzymatic and genetic analyses of α-galactosidase A. Enzymatic analysis was performed using the Dried Blood Filter Paper test (DBFP) described by Chamoles et al., with minor modifications [12]. The patient showed almost a normal value of α-galactosidase A activity in the blood; nevertheless, this value give no clear indication of FD because the subject affected is a female. As an X-linked genetic disorder, FD was first thought to affect only men and women were generally considered to be asymptomatic carriers. However, recent studies suggest that female heterozygotes for FD may still develop vital organ damage causing severe morbidity and mortality.

Genetic analysis was performed by high resolution melting (HRM) analysis on DNA samples, isolated from whole blood, using the Light Cycler 480 system [13]. PCR products presenting melting curves different in position or shape from those of the wild type control were sequenced to identify the suspected mutations. DNA sequencing has identified the presence of four previously reported mutations, combined in a heterozygote haplotype -10C>T (g.1170C>T), c.370-77_-81del (g.7188-7192del5), c.640-16A>G (g.10115A>G), c.1000-22C>T (g.10956C>T), mapping in promoter and regulatory intronic regions [14-18]. These intronic mutations could play both qualitative and quantitative roles in the transcription of the gene and in the translation of α-galactosidase A, a hypothesis strengthened in some cases by the presence of Gb3 and/or lyso-Gb3 in the blood and urine of patients [18]. It has been shown that regulatory intronic regions may regulate the splicing of the corresponding mRNA, and that mutations in these regions, or factors that act in trans with them, can cause genetic diseases [19,20].

After these findings, the patient underwent to enzyme replacement therapy with agalsidase alfa, the efficacy of which is due to its ability to reverse lysosomal glycosphingolipid accumulation. Due to the short period of treatment, to date patient’s symptoms have not improved but disease progression was arrested.

## Conclusions

In this paper, we reported the case of a 40-year-old female patient in which clinical history and specific instrumental findings suggested the diagnosis of FD. A genetic survey found four intronic mutations, in heterozygosis, in positions -10C>T (g.1170C>T), c.370-77_-81del (g.7188-7192del5), c.640-16A>G (g.10115A>G), c.1000-22C>T (g.10956C>T) in the GLA gene. These mutations have been observed in several patients with manifestations of FD characterized by not severe clinical picture, with symptoms affecting a specific organ, and that occur later in life because residual enzyme activity remains high enough to prevent organ damage in childhood and adolescence. In our patient multisystemic involvement and early onset of symptoms seems to suggest a classical form of FD, without any mutations in coding region of the GLA gene but presenting a combined heterozygote intronic haplotype. Our results and those recently reported in the literature support the idea that FD is not only caused by genetic alterations in the exons of GLA, but even by those occurring in regions of gene regulation [3]. It has been shown that aberrant splicing of the messenger is the cause of at least 15% of human genetic diseases, stressing the importance of studying also intronic regions of the genes involved [21].

Since non-coding regions are not routinely evaluated when sequencing the GLA gene, the occurence of intronic disease-causing lesions should encourage to analyze them and to perform, in patients with such mutations, further clinical and instrumental evaluations. This can lead to confirm diagnosis of FD in a higher number of patients, thus providing a more realistic estimation of the prevalence of FD in general population.

## Consent

Written informed consent was obtained from the patient for publication of this case report and any accompanying images. A copy of the written consent is available for review by the Series Editor of this journal.

The study was approved by the University Hospital Ethics Committee.

## Abbreviations

FD, Anderson-Fabry disease; GLA, α-galactosidase A; Gb3, Globotriaosylceramide; TIA, Transient ischemic attack; LV, Left ventricular; MRI, Magnetic resonance imaging; MT2RT, Myocardial T2 relaxation time; HRM, High resolution melting.

## Competing interests

The authors declare that they have no competing interests.

## Authors’ contributions

AP performed instrumental and clinical analyses; MI performed instrumental and clinical analyses; CZ performed the sequence analysis; GA carried out the enzymatic analysis; PC performed the genetic analysis; RA participated in the review of the manuscript; FI participated in acquisition and interpretation of data; GD coordinated the study and obtained the informed consent and ethics approval to conduct the study. All authors read and approved the final manuscript.

## Pre-publication history

The pre-publication history for this paper can be accessed here:

http://www.biomedcentral.com/1471-2261/12/39/prepub
